# EXPLORING PROMISING PRACTICE MODELS FOR HOUSING OLDER PERSONS EXPERIENCINGHOMELESSNESS

**DOI:** 10.1093/geroni/igz038.3283

**Published:** 2019-11-08

**Authors:** Christine Walsh, Sarah L Canham, Joe Humphries, Victoria F Burns, Natasha Dharshi, Tamara Sussman, Jackson Hangar

**Affiliations:** 1 University of Calgary, Calgary, Alberta, Canada; 2 Simon Fraser University, Vancouver, British Columbia, Canada; 3 Simon Fraser University, Burnaby, British Columbia, Canada; 4 McGill University, Montreal, Quebec, Canada

## Abstract

The numbers of older persons experiencing homelessness (OPEH) is on the rise globally Yet housing and shelter options that support the varied and complex needs of this population are scarce. In order to understand effective solutions for housing OPEH, it is critical to explore promising practices that support aging in the right place for OPEH. In an effort to inform this critical gap, 100 OPEH and service providers were purposefully selected and invited to attend one of three World Café workshops held in three major urban cities in Canada: Vancouver, Calgary, and Montréal. Participants engaged in facilitated discussions aimed at supporting knowledge exchange and generating dialogue about gaps, opportunities and promising local housing options. Thematic analyses of audiotaped deliberations revealed three themes: 1) The limited nature of current housing options and programs in each locality; 2) The importance of supporting integrative housing models that increase access to formal health and social support staff, transportation, and income supports; and 3) The significance of supporting sustainability, by conducting regular program evaluations, increasing public awareness of homelessness issues, and involving multi-sector stakeholders. Findings highlight how meeting the unique health and psychosocial needs of OPEH requires a nuanced understanding of the development, design, and sustainability of effective housing options. World Café dialogues revealed that identifying and sustaining existing promising practice models provides an avenue to supporting aging in the right place for OPEH.

